# Supplementary material for: Comparing acoustic and radar deterrence methods as mitigation measures to reduce human-bat impacts and conservation conflicts
Source: PLoS One. 2020 Feb 13;15(2):e0228668. doi: 10.1371/journal.pone.0228668 (PMC7018087; doi:10.1371/journal.pone.0228668)
Supplement: S1 Table — (DOCX) [file pone.0228668.s001.docx]

**S1 Table. Near infrared (NIR) bat pass count data.** The number of bat passes counted crossing the field of view on a near infrared video footage and temperature (°C) at 14 sites during four ten-minute time blocks (A-C), alternated with deterrent treatments and a silent control, including an ultrasound only treatment, an ultrasound and radar treatment and a radar only treatment.

| **Site** | **Treatment** | **Time block** | **Temp** (°C) | **Bat pass count** |
| --- | --- | --- | --- | --- |
| A | Ultrasound + Radar | A | 13 | 4 |
| A | Ultrasound | B | 14 | 17 |
| A | Radar | C | 13 | 70 |
| A | Control | D | 13 | 137 |
| B | Control | A | 14 | 57 |
| B | Ultrasound + Radar | B | 14 | 28 |
| B | Ultrasound | C | 14 | 28 |
| B | Radar | D | 14 | 53 |
| C | Ultrasound | A | 17 | 3 |
| C | Control | B | 16 | 6 |
| C | Radar | C | 17 | 15 |
| C | Ultrasound + Radar | D | 16 | 3 |
| D | Radar | A | 15 | 345 |
| D | Ultrasound + Radar | B | 14 | 22 |
| D | Ultrasound | C | 15 | 34 |
| D | Control | D | 14 | 50 |
| E | Ultrasound + Radar | A | 15 | 4 |
| E | Radar | B | 15 | 29 |
| E | Control | C | 15 | 11 |
| E | Ultrasound | D | 16 | 5 |
| F | Control | A | 14 | 5 |
| F | Ultrasound | B | 15 | 2 |
| F | Ultrasound + Radar | C | 14 | 20 |
| F | Radar | D | 15 | 16 |
| G | Ultrasound | A | 14 | 2 |
| G | Ultrasound + Radar | B | 12 | 6 |
| G | Control | C | 12 | 5 |
| G | Radar | D | 13 | 5 |
| H | Radar | A | 12 | 134 |
| H | Ultrasound | B | 13 | 5 |
| H | Ultrasound + Radar | C | 12 | 6 |
| H | Control | D | 12 | 21 |
| I | Ultrasound + Radar | A | 12 | 15 |
| I | Control | B | 11 | 38 |
| I | Radar | C | 12 | 9 |
| I | Ultrasound | D | 13 | 1 |

**S1 Table continued.**

| **Site** | **Treatment** | **Time Block** | **Temp** | **Bat pass count** |
| --- | --- | --- | --- | --- |
| J | Control | A | 12 | 66 |
| J | Radar | B | 13 | 61 |
| J | Ultrasound | C | 13 | 10 |
| J | Ultrasound + Radar | D | 12 | 3 |
| K | Ultrasound | A | 14 | 14 |
| K | Radar | B | 13 | 90 |
| K | Ultrasound + Radar | C | 13 | 2 |
| K | Control | D | 12 | 33 |
| L | Radar | A | 13 | 5 |
| L | Ultrasound | B | 14 | 1 |
| L | Control | C | 12 | 50 |
| L | Ultrasound + Radar | D | 12 | 1 |
| M | Ultrasound + Radar | A | 16 | 5 |
| M | Control | B | 16 | 29 |
| M | Ultrasound | C | 16 | 7 |
| M | Radar | D | 16 | 18 |
| N | Control | A | 14 | 62 |
| N | Ultrasound + Radar | B | 14 | 22 |
| N | Radar | C | 15 | 12 |
| N | Ultrasound | D | 17 | 8 |
