# Supplementary material for: Comparing acoustic and radar deterrence methods as mitigation measures to reduce human-bat impacts and conservation conflicts
Source: PLoS One. 2020 Feb 13;15(2):e0228668. doi: 10.1371/journal.pone.0228668 (PMC7018087; doi:10.1371/journal.pone.0228668)
Supplement: S2 Table — (DOCX) [file pone.0228668.s002.docx]

**S2 Table. *Pipistrellus pygmaeus* pass count data.** The number of *Pipistrellus pygmaeus* passes recorded at six sites during four ten-minute time blocks (A-C), alternated with deterrent treatments and silent control, including an ultrasound only treatment, an ultrasound and radar treatment and a radar only treatment.

| **Site** | **Treatment** | **Time block** | **Bat pass count** |
| --- | --- | --- | --- |
| A | Ultrasound | 1 | 10 |
| A | Radar | 2 | 174 |
| A | Ultrasound+Radar | 3 | 106 |
| A | Control | 4 | 215 |
| B | Control | 1 | 67 |
| B | Ultrasound+Radar | 2 | 57 |
| B | Ultrasound | 3 | 45 |
| B | Radar | 4 | 42 |
| C | Ultrasound+Radar | 1 | 9 |
| C | Radar | 2 | 43 |
| C | Control | 3 | 10 |
| C | Ultrasound | 4 | 25 |
| D | Ultrasound | 1 | 14 |
| D | Ultrasound+Radar | 2 | 15 |
| D | Control | 3 | 12 |
| D | Radar | 4 | 51 |
| E | Control | 1 | 52 |
| E | Radar | 2 | 52 |
| E | Ultrasound | 3 | 4 |
| E | Ultrasound+Radar | 4 | 2 |
| F | Ultrasound+Radar | 1 | 125 |
| F | Control | 2 | 170 |
| F | Ultrasound | 3 | 108 |
| F | Radar | 4 | 110 |
