# Supplementary material for: Comparing acoustic and radar deterrence methods as mitigation measures to reduce human-bat impacts and conservation conflicts
Source: PLoS One. 2020 Feb 13;15(2):e0228668. doi: 10.1371/journal.pone.0228668 (PMC7018087; doi:10.1371/journal.pone.0228668)
Supplement: S3 Table — (DOCX) [file pone.0228668.s003.docx]

**S3 Table. *Pipistrellus pipistrellus* pass count data.** The number of *Pipistrellus pipistrellus* passes recorded at six sites during four ten-minute time blocks (A-C), alternated with deterrent treatments and silent control, including an ultrasound only treatment, an ultrasound and radar treatment and a radar only treatment.

| **Site** | **Treatment** | **Time block** | **Bat pass count** |
| --- | --- | --- | --- |
| A | Ultrasound | A | 6 |
| A | Radar | B | 59 |
| A | Ultrasound+Radar | C | 17 |
| A | Control | D | 59 |
| B | Control | A | 60 |
| B | Ultrasound+Radar | B | 2 |
| B | Ultrasound | C | 4 |
| B | Radar | D | 14 |
| C | Ultrasound+Radar | A | 0 |
| C | Radar | B | 7 |
| C | Control | C | 22 |
| C | Ultrasound | D | 32 |
| D | Ultrasound | A | 51 |
| D | Ultrasound+Radar | B | 14 |
| D | Control | C | 45 |
| D | Radar | D | 40 |
| E | Control | A | 45 |
| E | Radar | B | 70 |
| E | Ultrasound | C | 24 |
| E | Ultrasound+Radar | D | 8 |
| F | Ultrasound+Radar | A | 12 |
| F | Control | B | 18 |
| F | Ultrasound | C | 3 |
| F | Radar | D | 6 |
