# Supplementary material for: Comparing acoustic and radar deterrence methods as mitigation measures to reduce human-bat impacts and conservation conflicts
Source: PLoS One. 2020 Feb 13;15(2):e0228668. doi: 10.1371/journal.pone.0228668 (PMC7018087; doi:10.1371/journal.pone.0228668)
Supplement: S4 Table — (DOCX) [file pone.0228668.s004.docx]

**S4 Table. *Myotis* species pass count data.** The number of *Myotis* species passes recorded at six sites during four ten-minute time blocks (A-C), alternated with deterrent treatments and silent control, including an ultrasound only treatment, an ultrasound and radar treatment and a radar only treatment.

| **Site** | **Treatment** | **Time block** | **Bat pass count** |
| --- | --- | --- | --- |
| A | Ultrasound | 1 | 0 |
| A | Radar | 2 | 2 |
| A | Ultrasound+Radar | 3 | 49 |
| A | Control | 4 | 18 |
| B | Control | 1 | 65 |
| B | Ultrasound+Radar | 2 | 57 |
| B | Ultrasound | 3 | 69 |
| B | Radar | 4 | 47 |
| C | Ultrasound+Radar | 1 | 0 |
| C | Radar | 2 | 7 |
| C | Control | 3 | 7 |
| C | Ultrasound | 4 | 8 |
| D | Ultrasound | 1 | 4 |
| D | Ultrasound+Radar | 2 | 22 |
| D | Control | 3 | 3 |
| D | Radar | 4 | 1 |
| E | Control | 1 | 3 |
| E | Radar | 2 | 4 |
| E | Ultrasound | 3 | 5 |
| E | Ultrasound+Radar | 4 | 3 |
| F | Ultrasound+Radar | 1 | 5 |
| F | Control | 2 | 1 |
| F | Ultrasound | 3 | 8 |
| F | Radar | 4 | 0 |
