# Supplementary material for: Comparing acoustic and radar deterrence methods as mitigation measures to reduce human-bat impacts and conservation conflicts
Source: PLoS One. 2020 Feb 13;15(2):e0228668. doi: 10.1371/journal.pone.0228668 (PMC7018087; doi:10.1371/journal.pone.0228668)
Supplement: S5 Table — (DOCX) [file pone.0228668.s005.docx]

**S5 Table. Bat feeding buzz count data.** The number of feeding buzzes recorded at six sites during four ten-minute time blocks (A-C), alternated with deterrent treatments and silent control, including an ultrasound only treatment, an ultrasound and radar treatment and a radar only treatment.

| **Site** | **Treatment** | **Time block** | **Feeding buzzes** |
| --- | --- | --- | --- |
| A | Ultrasound | A | 1 |
| A | Radar | B | 75 |
| A | Ultrasound+Radar | C | 48 |
| A | Control | D | 122 |
| B | Control | A | 25 |
| B | Ultrasound+Radar | B | 12 |
| B | Ultrasound | C | 12 |
| B | Radar | D | 11 |
| C | Ultrasound+Radar | A | 6 |
| C | Radar | B | 27 |
| C | Control | C | 19 |
| C | Ultrasound | D | 15 |
| D | Ultrasound | A | 0 |
| D | Ultrasound+Radar | B | 3 |
| D | Control | C | 4 |
| D | Radar | D | 3 |
| E | Control | A | 12 |
| E | Radar | B | 6 |
| E | Ultrasound | C | 3 |
| E | Ultrasound+Radar | D | 2 |
| F | Ultrasound+Radar | A | 39 |
| F | Control | B | 31 |
| F | Ultrasound | C | 14 |
| F | Radar | D | 24 |
